# Supplementary material for: An Erg-driven transcriptional program controls B cell lymphopoiesis
Source: Nat Commun. 2020 Jun 15;11:3013. doi: 10.1038/s41467-020-16828-y (PMC7296042; doi:10.1038/s41467-020-16828-y)
Supplement: Supplementary file 1 — Supplementary Information [file 41467_2020_16828_MOESM1_ESM.pdf]

An Erg-driven transcriptional program controls B cell lymphopoiesis

Ng et al.

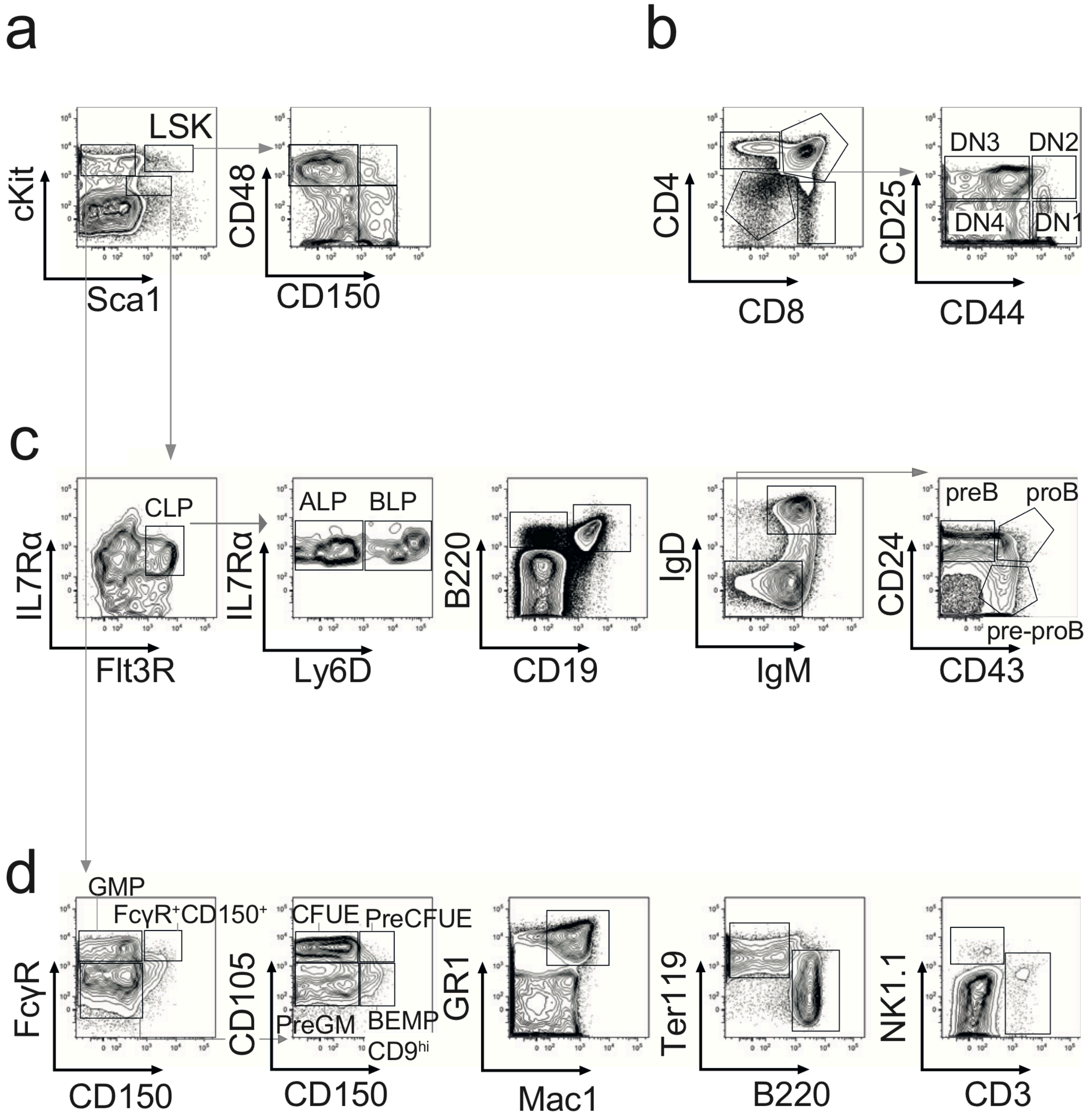

Supplementary Figure 1. Representative flow cytometry plots indicating gating strategies for analysis of hematopoietic cell populations. a. Bone marrow LSK cells, **b.** thymus sub-populations, **c.** bone marrow B lineage cells and **d.** bone marrow myeloid cell populations in *Erg*$^{KI/+}$ mice. The cell surface markers and definitions of cell populations used are provided in **Supplementary Table 1**.

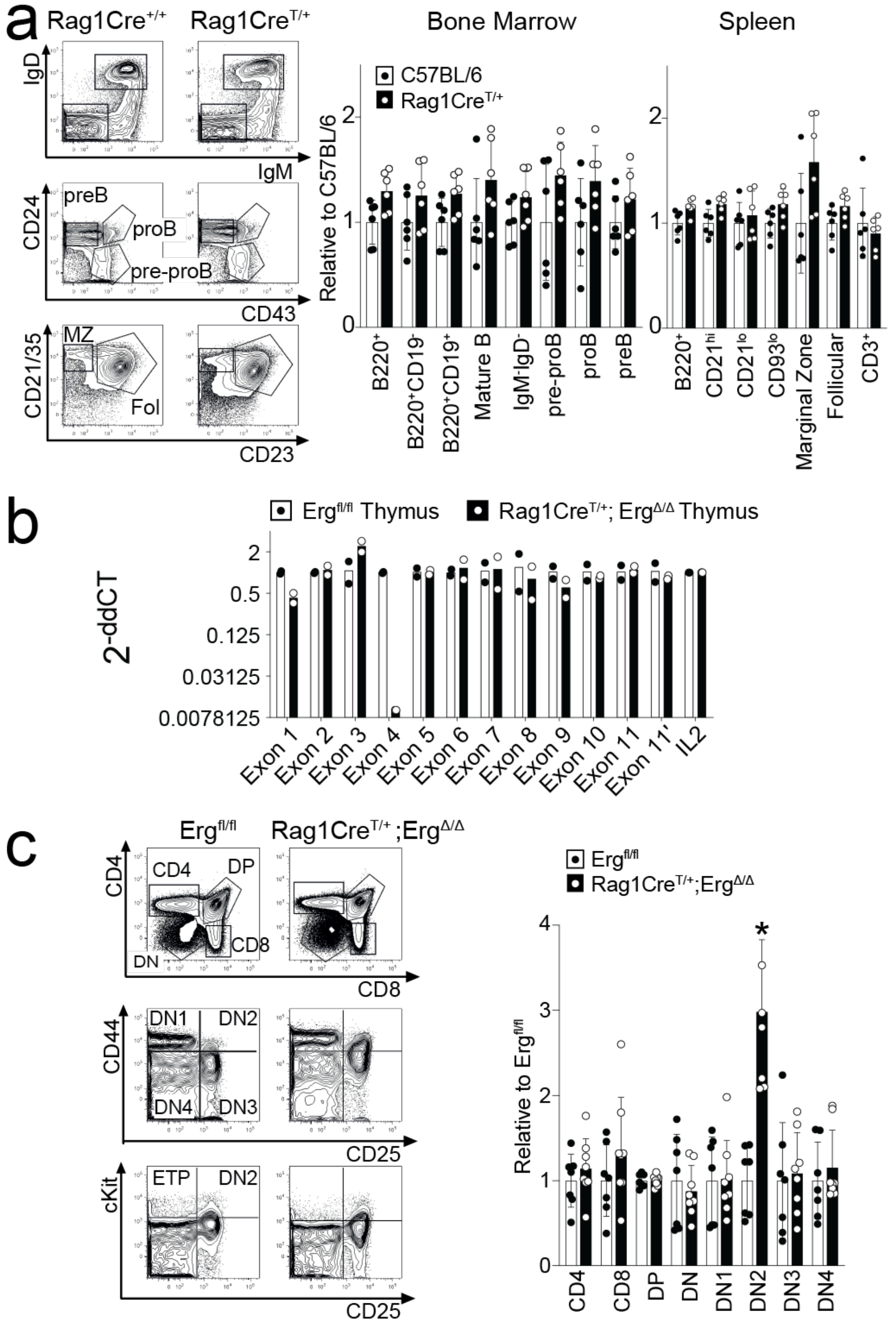

Supplementary Figure 2. B lymphopoiesis in *Rag1Cre*$^{T/+}$ mice and T lymphopoiesis in *Rag1Cre*$^{T/+}$;*Erg*$^{\Delta/\Delta}$ mice. a. Representative flow cytometry plots (left panels) of *Rag1Cre*$^{T/+}$ bone marrow and spleen cells. The IgM/IgD profile is from B220^{+} bone marrow cells (top panel), the CD24/CD43 profiles from B220^{+}IgM^{-}IgD^{-} bone marrow cells (middle panel) and the CD21/CD23 profiles from B220^{+}CD93^{lo} spleen cells (bottom panel). Ratio of *Rag1Cre*$^{+/+}$ and *Rag1Cre*$^{T/+}$ B lymphoid cells shown relative to *Rag1Cre*$^{+/+}$ controls in bone marrow and spleen with mean±S.D. shown (right panels). No significant differences were observed between the two genotypes for any

population by Student's two-tailed unpaired t-test corrected using Holm's modification for multiple testing (n=6 mice per genotype). See source data file for individual P_{adj} values. **b.** Quantitative genomic PCR on DNA from *Erg*$^{fl/fl}$ (n=2 biologically independent samples) and *Rag1Cre*$^{T/+}$*;Erg*$^{\Delta/\Delta}$ (n=2 biologically independent samples) thymocytes using primers spanning individual *Erg* exons [1] demonstrating efficient exon 4 deletion in *Rag1Cre*$^{T/+}$*;Erg*$^{\Delta/\Delta}$ thymocytes by $2^{-\Delta\Delta CT}$ method normalised to IL2 receptor and Erg exon 1. **c.** Representative flow cytometry plots (left panels) from *Erg*$^{fl/fl}$ and *Rag1Cre*$^{T/+}$*;Erg*$^{\Delta/\Delta}$ thymi identifying the specific cell populations indicated with the mean±S.D. of *Erg*$^{fl/fl}$ (n=7) and *Rag1Cre*$^{T/+}$*;Erg*$^{\Delta/\Delta}$ mice (n=8) shown relative to the mean of *Erg*$^{fl/fl}$ controls (right panel). No significant differences were observed other that in the DN2 population (*P* = 7.9e-5 by Student's two-tailed unpaired t-test. Source data are provided in the Source Data file.

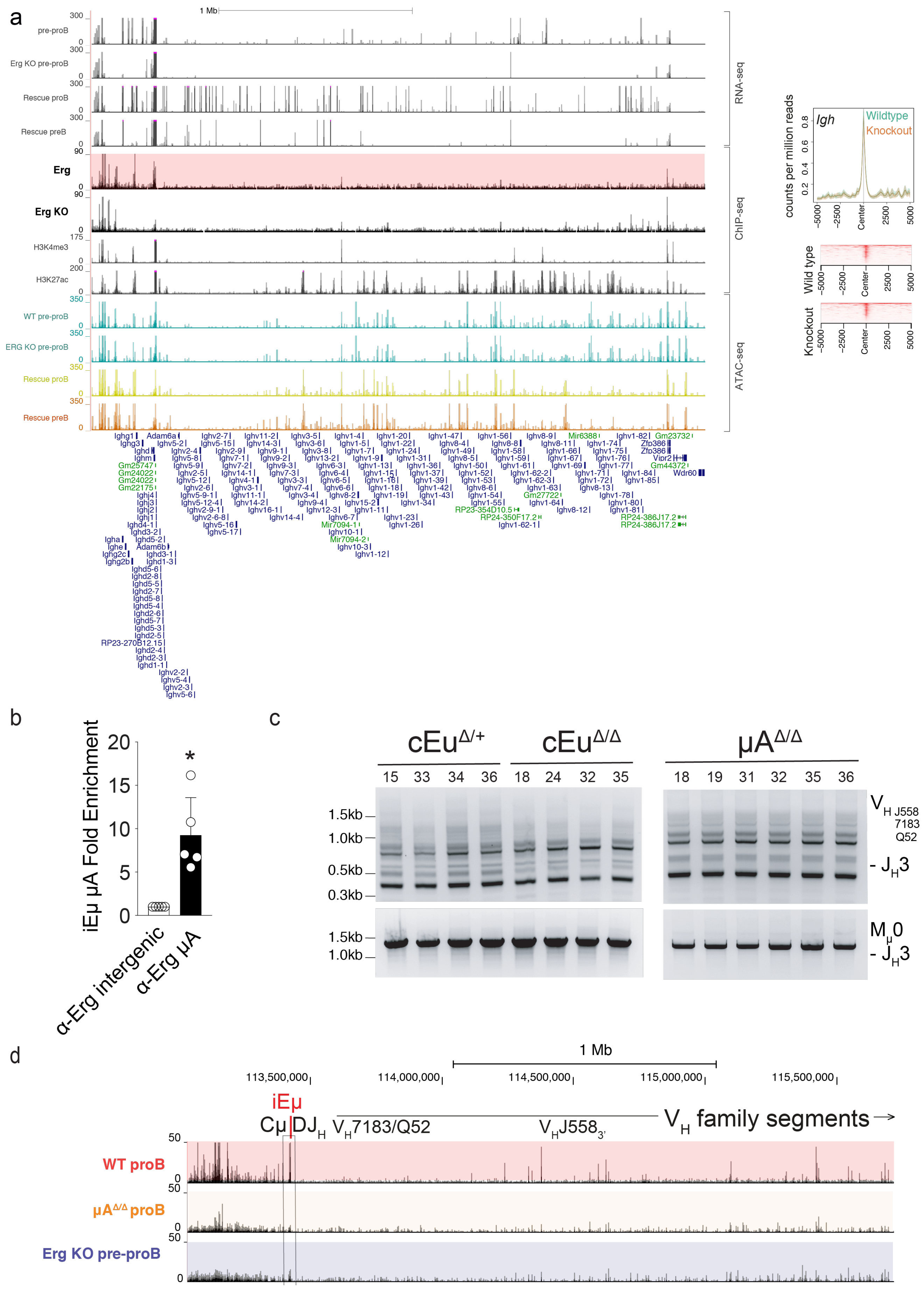

Supplementary Figure 3. In vivo Erg binding to the *Igh* locus and iEμ enhancer.

a. *Igh* locus with representative RNA-seq tracks shown for $Erg^{fl/fl}$ and $Rag1Cre^{T/+};Erg^{\Delta/\Delta}$ (Erg KO) pre-proB cells, and Erg deficient proB and preB cells in $Rag1Cre^{T/+};Erg^{\Delta/\Delta};IgH^{VH10tar/+}$mice rescued with a functionally rearranged immunoglobulin heavy chain allele (Rescue proB, Rescue preB). ChIP-seq tracks for Erg in wild-type proB cells (pink shading) and $Rag1Cre^{T/+};Erg^{\Delta/\Delta}$ thymus cells (Erg KO) to control for sites of non-Erg ChIP binding to DNA. H3K4me3 and H3K27ac ChIP-seq tracks in wild-type proB cells shown. Chromatin accessibility by ATAC-seq (blue) in WT and Erg KO pre-proB cells, and $Rag1Cre^{T/+};Erg^{\Delta/\Delta};IgH^{VH10tar/+}$ *Erg* deficient proB (yellow, Rescue proB) and preB (orange, Rescue preB) cells rescued with a functionally rearranged immunoglobulin heavy chain allele. **b.** Erg binding to iEμ containing the μA element by ChIP-PCR showing fold-enrichment (mean±S.D.) in wild type proB cells normalised to a negative intergenic region and ChIP input control using the $2^{-\Delta\Delta CT}$ method (n=5 biological replicates, * P=2.77e-3 by Student's two-tailed unpaired t-test). **c.** Genomic PCR using degenerate primers to *Igh* locus V_H558, V_H7183, V_HQ52 segments for detection of V_H-to-DJ_H (top panel) and Mu0 loading controls (bottom panel) in cEμ$^{\Delta/+}$, cEμ$^{\Delta/\Delta}$ and μA$^{\Delta/\Delta}$ B220^{+} splenocytes. Representative of two independent experiments. **d.** Erg binding to the 3' *Igh* region in C57BL/6 (WT) and μA$^{\Delta/\Delta}$ proB cells and $Rag1Cre^{T/+};Erg^{\Delta/\Delta}$ (Erg KO) pre-proB cells by ChIP-seq. Source data are provided in the Source Data file.

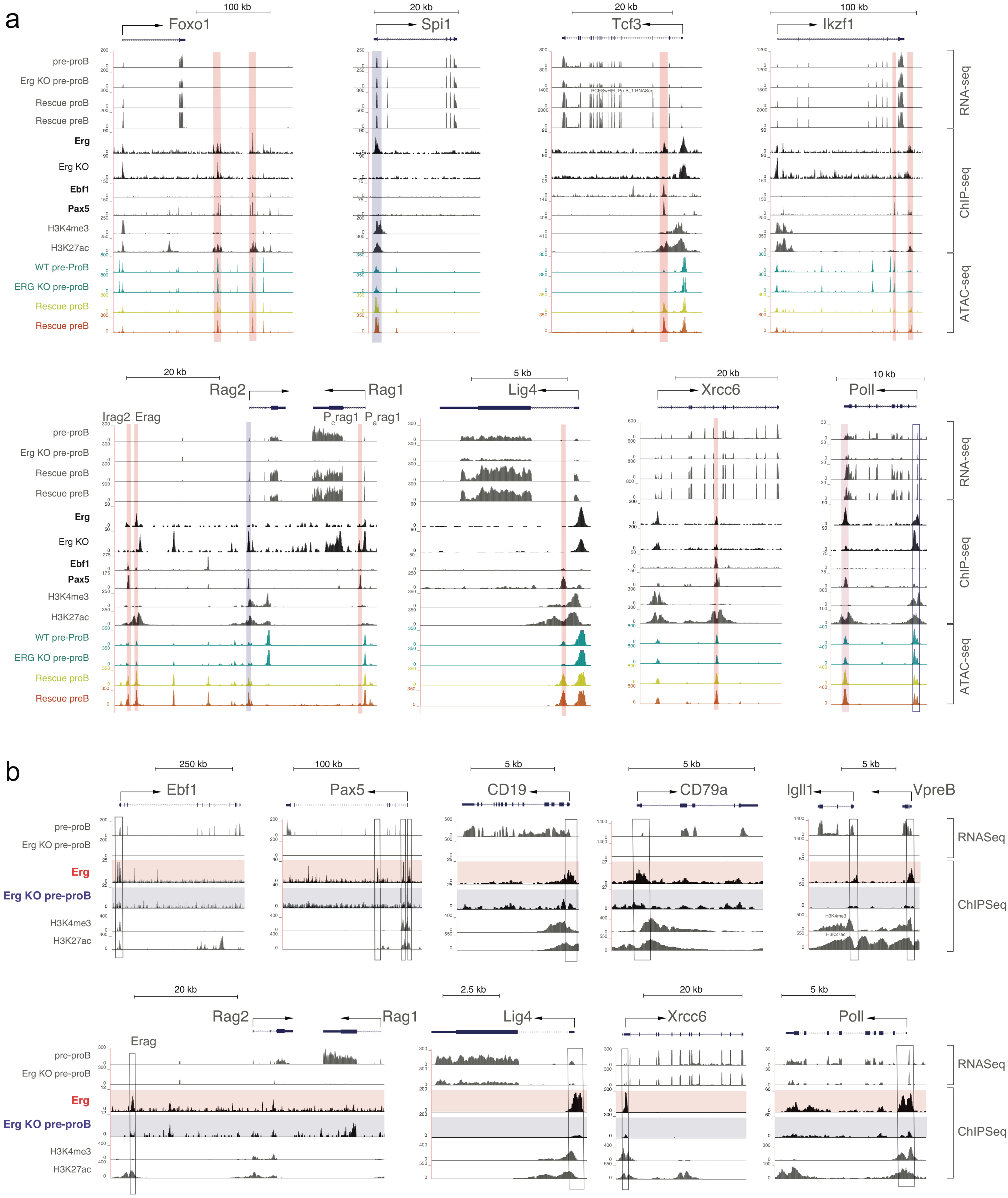

Supplementary Figure 4. RNA-seq and Erg, Ebf1 and Pax5 binding and chromatin accessibility at selected gene loci. a. RNA-seq gene expression at gene loci, with Erg, Ebf1, and Pax5 binding, H3K4me3 promoter mark, H3K27ac promoter and enhancer mark by ChIP-seq, and ATAC-seq in

Erg$^{fl/fl}$ pre-proB cells (pre-proB), *Rag1Cre*$^{T/+;}$*Erg*$^{\Delta/\Delta}$ pre-proB (Erg KO pre-proB), and Erg deficient proB and preB cells in *Rag1Cre*$^{T/+;}$*Erg*$^{\Delta/\Delta}$*;IgH*$^{VH10tar/+}$mice rescued with a functionally rearranged *Igh* allele (Rescue proB, Rescue preB). Erg, Ebf1 and/or Pax5 binding promoter region (blue shading). Erg, Ebf1 and/or Pax5 binding to enhancer regions (pink shading). **b.** RNA-seq at B cell gene loci in *Erg*$^{fl/fl}$ pre-proB cells (pre-proB) and *Rag1Cre*$^{T/+;}$*Erg*$^{\Delta/\Delta}$ pre-proB (Erg KO pre-proB), with Erg binding by ChIP-seq in C57BL/6 proB cells (Erg) using *Rag1Cre*$^{T/+;}$*Erg*$^{\Delta/\Delta}$ pre-proB cells (Erg KO pre-proB) as a negative control for sites of non-Erg ChIP binding to DNA. H3K4me3 promoter mark and H3K27ac promoter and enhancer mark are also shown. Open boxes indicate Erg binding to cis-regulatory promoter or enhancer regions.

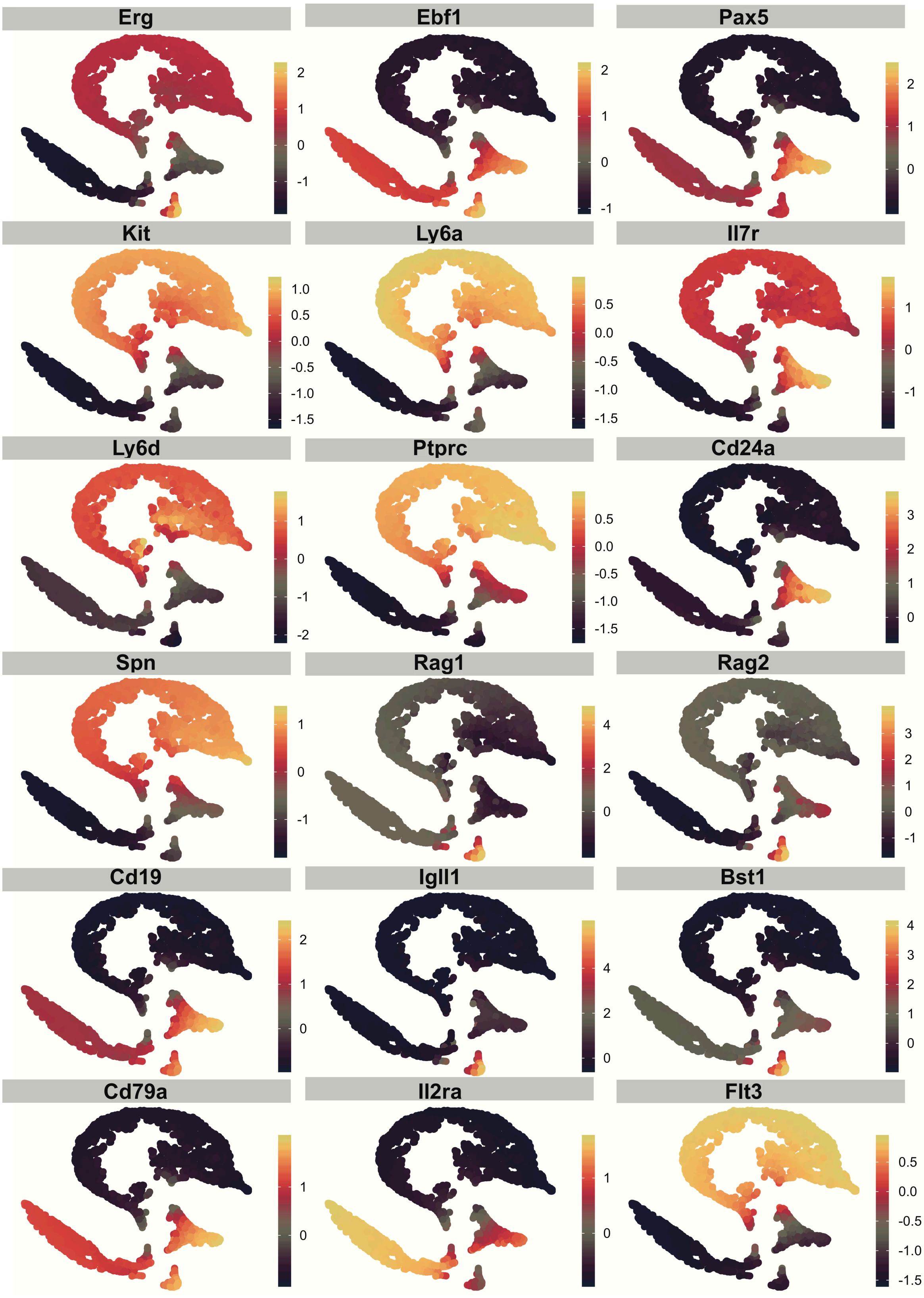

Supplementary Figure 5. Single cell RNA-seq analysis of B lymphoid populations. Single cell RNA-seq analysis (3297 cells, GSE114793) t-distributed stochastic neighbour embedding (tSNE) plots of CLP, pre-proB and $CD19^+$ proB and preB populations demonstrating the imputed expression of B lineage genes shown with interaction recovered using the MAGIC algorithm [2].

Supplementary Tables

Supplementary Table 1. Immunophenotype of hematopoietic cell populations

	Immunophenotype	Reference
Bone marrow		
LSK	Lineage$^-$ Sca-1$^+$Kit$^+$	[3, 4]
LT-HSC, CD150$^+$CD48$^-$	Lineage$^-$ Sca-1$^+$Kit$^+$CD150$^+$CD48$^-$	[5]
ST-HSC, CD150$^+$CD48$^+$	Lineage$^-$ Sca-1$^+$Kit$^+$CD150$^+$CD48$^+$	
MPP, CD150$^-$CD48$^+$	Lineage$^-$ Sca-1$^+$Kit$^+$CD150$^-$CD48$^+$	
PreGM	Lineage$^-$ IL7Rα^-cKit$^+$Sca1$^-$CD150$^-$ Endoglin$^-$FcγRII/III$^-$	[6, 7, 8]
GMP	Lineage$^-$ IL7Rα^-cKit$^+$Sca1$^-$CD150$^-$Endoglin$^-$ FcγRII/III$^-$	[9]
BEMP & CD9^{hi}	Lineage$^-$ IL7Rα^-cKit$^+$Sca1$^-$CD150$^+$Endoglin$^-$ FcγRII/III$^-$	[7]
PreCFUE	Lineage$^-$ IL7Rα^-cKit$^+$Sca1$^-$ CD150$^+$Endoglin$^+$FcγRII/III$^-$	[8]
CFUE	Lineage$^-$ IL7Rα^-cKit$^+$Sca1$^-$CD150$^-$ Endoglin$^+$FcγRII/III$^-$	[8]
CLP	Lineage$^-$ IL7Rα^+Flt3$^+$cKit^{lo}Sca1^{lo}	[10]
ALP	Lineage$^-$ IL7Rα^+Flt3$^+$cKit^{lo}Sca1^{lo}Ly6D$^-$	[11]
BLP	Lineage$^-$ IL7Rα^+Flt3$^+$cKit^{lo}Sca1^{lo}Ly6D$^+$	
pre-proB (Hardy Fraction A-to-B)	B220$^+$IgM$^-$IgD$^-$NK1.1$^-$CD11c$^-$CD19$^-$CD43$^+$CD24$^-$	[12, 13, 14, 15, 16, 17]
proB (Hardy Fraction C)	B220$^+$IgM$^-$IgD$^-$ NK1.1$^-$CD11c$^-$ CD19$^+$CD43$^+$CD24$^+$	
preB (Hardy Fraction D)	B220$^+$IgM$^-$IgD$^-$ NK1.1$^-$CD11c$^-$CD19$^+$CD43$^-$ CD24$^+$	
Immature B (Hardy Fraction E)	B220$^+$CD19$^+$IgM$^+$IgD$^-$	[12]
Mature recirculating B (Hardy Fraction F)	B220$^+$CD19$^+$IgM$^+$IgD$^+$	
Thymus		
DP	CD4$^+$CD8$^+$	
DN1	CD4$^-$CD8$^-$CD25$^-$CD44$^+$	[18]
DN2	CD4$^-$CD8$^-$CD25$^+$CD44$^+$	
DN3	CD4$^-$CD8$^-$CD25$^+$CD44$^-$	
DN4	CD4$^-$CD8$^-$CD25$^-$CD44$^-$	
Spleen		
Marginal Zone	B220$^+$CD19$^+$CD93^{lo}CD21/35^{hi}CD23^{lo}	[19, 20]
Follicular	B220$^+$ CD19$^+$CD93^{lo}CD21/35^{med}CD23^{hi}	

Supplementary Table 2. Peripheral blood counts in *Rag1Cre*$^{T/+}$;*Erg*$^{\Delta/\Delta}$ mice.

Genotype	RBC x10^{12}/L	Platelets x10^9/L	WBC x10^9/L	Neutrophil x10^9/L	Lymphocyte x10^9/L	Monocyte x10^9/L	Eosinophil x10^9/L
Erg$^{fl/fl}$ (n= 32)	11.03 ± 0.48	1038 ± 192	9.07 ± 1.64	1.00 ± 0.90	7.52 ± 1.66	0.18 ± 0.15	0.21 ± 0.11
Rag1Cre$^{T/+}$;*Erg*$^{\Delta/\Delta}$ (n=25)	10.73 ± 1.85	1256 ± 204	4.82 ± 1.72 *	1.09 ± 0.82	3.22 ± 1.12 *	0.22 ± 0.17	0.24 ± 0.06

Blood was collected into EDTA and differential cell counts performed using an ADVIA 120 Hematology System. RBC, red blood cells; WBC, white blood cells. * *Padj* $< 10^{-11}$ by Student's two-tailed unpaired t-test corrected for multiple testing by Benjamini-Hochberg procedure to control for false discovery rate.

Supplementary Table 3. Primers and PCR reactions

	Primer 1	Primer 2	Expected sizes
Erg[i]	WA972 5'-GGTGAGGTCTCTTCCTGAACC-3' common forward	WA974 5'-TTGGATCCTCAGAATCTACCG-3' exon 4 reverse<br>WA1092 5'- TTATCCTACCTGCCCCTGGT-3' 3' exon 4 reverse	Wildtype 206bp<br>Targeted 231bp<br>Floxed 355bp
V_H-to-DJ_H[ii]	VH558 5'-CGAGCTCTCCARCACAGCCTWCATGCARCTCARC-3'<br>VQ52 5'-CGGTACCAGACTGARCATCASCAAGGACAAYTCC-3'<br>VH7183 5'-CGGTACCAAGAASAMCCTGTWCCTGCAAATGASC-3' [21]	J3 5'-GTCTAGATTCTCACAAGAGTCCGATAGACCCTGG-3'	See **Figure 2.**
D_H-to-J_H[iii]	DHL 5'-GGAATTCGMTTTTTGTSAAGGGATCTACTACTGTG-3' [21]	J3 5'-GTCTAGATTCTCACAAGAGTCCGATAGACCCTGG-3'	See **Figure 2.**
Mu0[ii]	Mu0 5'-CCGCATGCCAAGGCTAGCCTGAAAGATTACC-3' [21]	J3 5'-GTCTAGATTCTCACAAGAGTCCGATAGACCCTGG-3'	Germline 1,259bp
VκD[iv]	VκD 5'-GGCTGCAGSTTCAGTGGCAGTGGRTCWGGRAC-3' [22]	Mar35 5'-AACACTGGATAAAGCAGTTTATGCCCTTTC-3' [23]	See **Figure 3.**
HEL-IgH[i]	VH10tarU 5'-GTCTCTGCAGGTGAGTCCTAACTTCT-3' [24]	VH10tarL 5'-CAACTATCCCTCCAGCCATAGGAT-3'	Wildtype 865bp<br>Knockin 302bp
IL2[i]	WA735 5'-CTAGGCCACAGAATTGAAAGATCT-3'	WA736 5'-GTAGGTGGAAATTCTAGCATCATCC-3'	
iEμ π/μA[vii]	iEμ 5'-TTTCGG/CTGAATCCTCAACT-3' forward<br>Chr12;113427394 to 113427413	iEμ 5'-GGTCATGTGGCAAGGCTATT-3' reverse<br>Chr12; 113427561 to 113427581	374bp
Erg –ve[vii]	Erg negative 5'-GGGAAACAACACCCTTCTCA-3' forward	Erg negative 5'-AATGTTGATCCTGCCAATCC-3' reverse	749bp

i. 94°C denaturation for 3 minutes, followed by 35 cycles at 94°C for 1 minute, 60°C for 30 seconds, 72°C for 1 minutes, and a final 5 minutes extension at 72°C.
ii. 94°C denaturation for 3 minutes, followed by 35 cycles at 94°C for 1 minute, 60°C for 30 seconds, 72°C for 2 minutes, and a final 5 minutes extension at 72°C.
iii. 94°C denaturation for 3 minutes, followed by 40 cycles of 1 minute at 94°C, 1 minute at 60°C, and 1 minute at 72°C, and a final extension step at 72°C for 10 min [25].
iv. 94°C denaturation for 3 minutes, followed by 27 cycles at 94°C for 1 minute, 60°C for 1 minute, 68°C for 3 minute, and a final 5 minute extension at 68°C [26].
v. 95°C denaturation for 3 minutes, followed by 35 cycles at 95°C for 30 seconds, 58°C for 40 seconds, 72°C for 45 seconds [27].
vi. 94°C denaturation for 5 min, followed by 30 cycles at 94°C for 30 seconds, annealing temperature 30 seconds, 72°C for 1 min, and a final 10 min extension at 72°C. The annealing temperature was held at 68ºC, 65ºC and 62ºC for five cycles each and 58ºC for 15 cycles [28].
vii. 95°C denaturation for 20 seconds, followed by 40 cycles at 95°C for 3 seconds, 60°C for 30 seconds.

Supplementary Table 4. Key Resources Table

REAGENT or RESOURCE			SOURCE	IDENTIFIER
Antibodies				
Anti-Erg			Santa Cruz Biotechnology	sc-354
Anti-Erg			Abcam	ab133264
Anti-Ebf1			Abcam	ab108369
Anti-Pax5			In-house	Clone: 1H9
Flow Cytometry Antibodies				
Antibodies	Fluorochrome	Concentration		
Ter119	APC-Cy7	1:150	BD Biosciences	Clone : Ly-76
CD41	PE	1:333	BD Biosciences	Clone : MWReg30
Gr1	PE-Cy7	1:333	BD Biosciences	Clone :Ly6G &Ly6C
Mac1	Alexa700	1:333	BD Biosciences	Clone : CD11b
NK1.1	PerCPCy5_5	1:333	BD Biosciences	Clone : N418
CD45R/B220	APC	1:333	BD Biosciences	Clone : RA3-6B2
CD19	Biotin	1:333	BD Biosciences	Clone : 1D3
CD3	PE	1:333	BD Biosciences	Clone : 17A2
CD4	APC	1:300	BD Biosciences	Clone : CK1.5
CD8a	PE	1:300	BD Biosciences	Clone : 53.6.7
Sca1 (Ly6A/E)	PE-Cy7	1:200	BD Biosciences	Clone : D7
cKit (CD117)	PerCP-Cy5.5	1:200	BD Biosciences	Clone : 2B8 or ACK4
CD150	APC	1:100	Biolegend	Clone : TC15-12F12.2
CD105	PE	1:100	BD Biosciences	Clone : MJ7/18
CD16/32	PE-Cy7	1:100	BD Biosciences	Clone ; 24G2
CD127	Biotin	1:100	eBioscience	Clone : A7R34
CD135	PE	1:50	Biolegend	Clone : A2F10
Ly6D	APC	1:1000	BD Biosciences	Clone : 49-H4
CD21/35	FITC	1:400	BD Biosciences	Clone : 7G6
CD23	PE-Cy7	1:400	BD Biosciences	Clone : B3B4
CD93	PerCP-Cy5.5	1:400	BD Biosciences	Clone : AA4.1
CD24	PerCP-Cy5.5	1:400	BD Biosciences	Clone : M1/69
CD43	PE-Cy7	1:400	BD Biosciences	Clone : S7
CD45.2	Biotin	1:400	BD Biosciences	Clone : S450-15-2
CD45.1	Alexa700	1:400	BD Biosciences	Clone : A20
IgM^b	APC-Cy7	1:150	BD Biosciences	Clone : AF6-78
IgD	PE	1:333	BD Biosciences	Clone : 11-26c.2a
CD138	PE	1:200	BD Biosciences	Clone : 281.2
IgG1	APC	1:200	BD Biosciences	Clone : X56
CD25	PerCP-Cy5.5	1:400	BD Biosciences	Clone : 3C7
CD44	PE-Cy7	1:300	BD Biosciences	Clone : IM7
Chemicals, Peptides, and Recombinant Proteins				
Fluorescein di β galactopyranoside			Molecular Probes/Invitrogen	Catalog # : F1179
Recombinant Murine Interleukin-7			Peprotech	Catalog # : 217-17
Recombinant Murine Interleukin-4			R&D Systems	Catalog #: 404-ML-010
Recombinant Murine Interleukin-5			R&D Systems	Catalog # : 405-ML-005
Cell Trace Violet			Life Technologies	Catalog # : C34557
Lipopolysacchride			Difco	
AffiniPure F(ab')$_2$ Fragment Goat Anti-Mouse IgM µ chain specific			Jackson Immunoresearch	Catalog # : 715-006-020
CD40L			In-house	[29]
Deposited Data				
Sequence data and analysis related to this paper			This paper	https://www.ncbi.nlm.nih.gov/geo/

RNA-seq	This paper	GSE132854
ChIP-seq	This paper	GSE132853
ATAC-seq	This paper	GSE132852
Hi-C	This paper	GSE133246
ChIP-seq Ebf1	[30]	GSM1296532, GSM1296537
ChIP-seq ProB Rag2_Input	[30]	GSM1296537
ChIP-seq Pax5	[31]	GSM932924
ChIP-seq Prob_Rag2_Input_2	[31]	GSM1145867
ChIP-seq H3K4me3	[32]	GSM2255547
ChIP-seq H3K27ac	[32]	GSM2255552
RNA-seq Ebf1 knockout	[33]	GSM2879293, GSM2879294, GSM2879295
RNA-seq Pax5 knockout	[33]	GSM2879296, GSM2879297, GSM2879298
RNA-seq wild-type	[33]	GSM2879299, GSM2879300, GSM2879301
scRNA-seq wild-type	[33]	GSE114793
Experimental Models: Organisms/Strains		
Mouse: $Flpe^{T/+}$	[34] Susan Dymecki, Harvard University	N/A
Mouse: $Rag1Cre^{T/+}$	[35] Terry Rabbitts, University of Leicester	N/A
Mouse: $IgH^{VH10tar}$	[36] Robert Brink, Garvan Institute of Medical Research	N/A
Oligonucleotides		
WA972	Geneworks	WA972 5'-GGTGAGGTCTCTTCCTGAACC-3' common forward
WA974	Geneworks	WA974 5'-TTGGATCCTCAGAATCTACCG-3' exon 4 reverse
WA1092	Geneworks	WA1092 5'-TTATCCTACCTGCCCCTGGT-3' 3' exon 4 reverse
VH558	Geneworks	VH558 5'-CGAGCTCTCCARCACAGCCTWCATGCARCTCARC-3'
VQ52	Geneworks	VQ52 5'-CGGTACCAGACTGARCATCASCAAGGACAAYTCC-3'
VH7183	Geneworks	VH7183 5'-CGGTACCAAGAASAMCCTGTWCCTGCAAATGASC-3'
J3		5'-GTCTAGATTCTCACAAGAGTCCGATAGACCCTGG-3'
DHL	Geneworks	DHL 5'-GGAATTCGMTTTTTGTSAAGGGATCTACTACTGTG-3'
Mu0	Geneworks	Mu0 5'-CCGCATGCCAAGGCTAGCCTGAAAGATTACC-3'

VκD	Geneworks	VκD 5'-GGCTGCAGSTTCAGTGGCAGTGGRTCWGGRAC-3'
Mar35	Geneworks	Mar35 5'-AACACTGGATAAAGCAGTTTATGCCCTTTC-3'
VH10tarU	Geneworks	VH10tarU 5'-GTCTCTGCAGGTGAGTCCTAACTTCT-3'
VH10tarL	Geneworks	VH10tarL 5'-CAACTATCCCTCCAGCCATAGGAT-3'
iEμ forward	Geneworks	iEμ 5'-TTTCGG/CTGAATCCTCAACT-3' forward
iEμ reverse	Geneworks	iEμ 5'-GGTCATGTGGCAAGGCTATT-3' reverse
Erg negative forward	Geneworks	Erg negative 5'-GGGAAACAACACCCTTCTCA-3' forward
Erg negative reverse	Geneworks	Erg negative 5'-AATGTTGATCCTGCCAATCC-3' reverse
Recombinant DNA		
$Erg^{tm1a(KOMP)Wtsi}$ targeting vector	KOMP Knockout Mouse Project	Project ID: CSD48771
MSCV-mEbf1	In-house	N/A
MSCV-mPax5	In-house	N/A
FISH probe: VhJ558 Distal Region, IgHv1-72, BAC RP23-230L2	Creative Bioarray	N/A
FISH probe: Vh7183 Proximal Region, IgH5-2, BAC RP23-404D8	Creative Bioarray	N/A
Software and Algorithms		
FlowJo	Tree Star	RRID:SCR_008520
R Studio	RStudio, Inc	RRID:SCR_000432
R	R Project for Statistical Computing	RRID:SCR_001905
R packages Rsubread, limma, edgeR, Rmagic, Rtsne, csaw, diffHic, InterationSet, Sushi	Bioconductor	RRID:SCR_001905
MACS2	Liu Lab, Harvard University	RRID:SCR_013291
Bowtie2	[37]	RRID:SCR_005476
deepTools	[38]	RRID:SCR_016366
R packages pheatmap viridis	CRAN	RRID:SCR_003005S
ImageJ	[39]	RRID:SCR_003070
Zen	Zeiss Microscopy	RRID:SCR_013672
Huygens Software	Scientific Volume Imaging	RRID:SCR_014237

Supplementary Methods

Genomic PCR. Genomic DNA was extracted using DirectPCR lysis reagent (Viagen) with proteinase K (Sigma-Aldrich) or the DNeasy minikit (Qiagen). 1µL of supernatant from murine tail samples lysed in 200µL or 50-100ng of genomic DNA were used for each reaction. Conditional *Erg* genomic deletion was detected using primers designed to detect the wild-type, floxed or deleted *Erg* alleles (**Supplementary Table 3**). Degenerate PCR primers for detection of genomic recombination across distal V_H558 or proximal V_H7183, V_HQ52 regions, the D_H region or Mu0 regions to J3 segments [21], and Vκ [22] were used as described, as were primers to detect TCR VβJ recombination [40, 41] and the $IgH^{VH10tar}$ allele [24] **(Supplementary Table 3)** [42] [25, 26, 28]. PCR products were separated by agarose gel electrophoresis and visualized with ethidium bromide staining. For quantitative genomic PCR using SYBR green (Life technologies), primers spanning individual *Erg* exons were used as described [1] and relative quantitation was performed using the $2^{-\Delta\Delta CT}$ method [43].

RNA-seq of primary B lymphoid samples. Total RNA was extracted using the RNeasy Plus minikit (Qiagen) from bone marrow B lymphoid populations sorted independently from two $Rag1Cre^{T/+};Erg^{\Delta/\Delta}$ and $Rag1Cre^{+/+};Erg^{fl/fl}$ mice at 7-10 weeks of age. Sequencing was performed on an Illumina Hi-Seq 2500 to generate 100bp paired-end reads. Two biological replicates were sequenced for each mouse strain and B cell development stage. Adapter sequences were removed using Trimgalore (https://github.com/FelixKrueger/TrimGalore). Reads were aligned to the mm10 mouse genome using STAR [44]. Genewise counts were obtained using featureCounts [45] with Rsubread's built-in Entrez Gene annotation [46]. Downstream analysis as conducted using edgeR 3.22.5 [47]. For each B cell stage, genes were filtered as non-expressed if they were assigned 0.5 counts per million mapped reads (CPM) in fewer than two libraries. Library sizes were TMM normalized and differential expression was assessed using quasi-likelihood F-tests [48]. Genes were called differentially expressed if they achieved a false discovery rate of 0.05 (**Supplementary Data 1**). For plotting purposes, counts were converted to Fragments Per Kilobase of transcript per Million mapped reads

(FPKM) using edgeR's rpkm function. These data have been deposited in Gene Expression Omnibus database (accession number GSE132854 [https://www.ncbi.nlm.nih.gov/geo/query/acc.cgi?acc=GSE132854]).

Chromatin Immunoprecipitation (ChIP). Chromatin immunoprecipitation was performed on $2x10^7$ cultured proB cells for Erg binding using the ab133264 antibody. Cells were cross-linked with 1% formaldehyde for 15 min at room temperature, terminated by the addition of 0.125M glycine. Cells were then lysed in 1% SDS, 10mM EDTA, 50mM Tris-HCl, pH8.0, and protease inhibitors. Lysates were sonicated in a Covaris ultrasonicator to achieve a mean DNA fragment size of 500 bp. Immunoprecipitation was performed using 10µg of antibodies for a minimum of 12h at 4°C in modified RIPA buffer (1% Triton X-100, 0.1% deoxycholate, 90mM NaCl, 10mM Tris-HCl, pH8.0 and protease inhibitors). An equal volume of protein A and G magnetic beads (Life Technologies) were used to bind the antibody and associated chromatin. Reverse crosslinking of DNA was performed at 65°C overnight with RNaseA digestion followed by DNA purification using QIAquick PCR purification kits (Qiagen). Immunoprecipitated DNA was analysed on an Applied Biosystems StepOnePlus System with SYBR green reagents for iEµ µA and intergenic negative control regions using specific primers as detailed in **Supplementary Table 3**. Relative ChIP PCR enrichment of the iEµ µA containing region in proB cells compared to input was performed and normalized to the intergenic negative control region using the $2^{-\Delta\Delta CT}$ method [43].

ChIP-seq. For sequencing analysis of immunoprecipitated DNA, DNA was quantified using the Qubit dsDNA HS Assay (Life Technologies). Library preparations were performed using the standard ThruPLEXTM-FD Prep Kit protocol (Rubicon Genomics) and size selected for 200–400bp fragments using Pippen Prep (Sage Science Inc.). Fragment sizes were confirmed using either the High Sensitivity DNA assay or the DNA 1000 kit and 2100 bioanalyzer (Agilent Technologies). Libraries were quantified with qPCR, normalized and pooled to 2nM before sequencing with single-end 75bp reads using standard protocols on the NextSeq (Illumina). DNA reads were adapter trimmed using Trimmomatic [49] and aligned to the GRCm38/mm10 build of the Mus musculus genome using the

BWA aligner [50]. Peaks were called using MACS2 [51] with default parameters to identify peaks using C17 antibody for Erg binding with *Rag1Cre*$^{T/+}$*;Erg*$^{\Delta/\Delta}$ thymocytes as a negative control to filter peaks not due to Erg binding or ab133264 antibody in *Rag1Cre*$^{T/+}$*;Erg*$^{\Delta/\Delta}$ pre-proB cells and *uA*$^{\Delta/\Delta}$ proB cells and wild-type proB cell controls, and were annotated to closest (peak start within 10kb from TSS) and overlapping genes using the R/Bioconductor package ChIPpeakAnno [52] (**Supplementary Data 2**). These data have been deposited in Gene Expression Omnibus database (accession number GSE132853 [https://www.ncbi.nlm.nih.gov/geo/query/acc.cgi?acc=GSE132853]). Publicly available FASTQ files for Ebf1 (GSM1296532, GSM1296537), Pax5 (GSM932924), H3K4me3 (GSM2255547) and H3K27Ac (GSM2255552) ChIP-seq experiments were aligned to the mm10 mouse reference genome (GRCm38, December 2011) using Rsubread [53]. Peak-calling was performed using MACS2 [51] against input FASTQ files (GSM1296537, GSM1145867). Coordinates for annotated immunoglobulin heavy chains were obtained from Ensemble/Biomart (accessed 6th March 2017) and coordinates for 3' regulatory region (3'RR) hypersensitivity regions, 3'αE, iEμ, were as published [54] [55] [56] [57].

Supplementary References

1. Loughran, S.J. *et al.* The transcription factor Erg is essential for definitive hematopoiesis and the function of adult hematopoietic stem cells. *Nat Immunol* **9**, 810-819 (2008).

2. van Dijk, D. *et al.* Recovering Gene Interactions from Single-Cell Data Using Data Diffusion. *Cell* **174**, 716-729 e727 (2018).

3. Osawa, M., Hanada, K., Hamada, H. & Nakauchi, H. Long-term lymphohematopoietic reconstitution by a single CD34-low/negative hematopoietic stem cell. *Science* **273**, 242-245 (1996).

4. Okada, S. *et al.* In vivo and in vitro stem cell function of c-kit- and Sca-1-positive murine hematopoietic cells. *Blood* **80**, 3044-3050 (1992).

5. Kiel, M.J. *et al.* SLAM family receptors distinguish hematopoietic stem and progenitor cells and reveal endothelial niches for stem cells. *Cell* **121**, 1109-1121 (2005).

6. Boiers, C. *et al.* Expression and role of FLT3 in regulation of the earliest stage of normal granulocyte-monocyte progenitor development. *Blood* **115**, 5061-5068 (2010).

7. Ng, A.P. *et al.* Characterization of thrombopoietin (TPO)-responsive progenitor cells in adult mouse bone marrow with in vivo megakaryocyte and erythroid potential. *Proc Natl Acad Sci U S A* **109**, 2364-2369 (2012).

8. Pronk, C.J. *et al.* Elucidation of the phenotypic, functional, and molecular topography of a myeloerythroid progenitor cell hierarchy. *Cell Stem Cell* **1**, 428-442 (2007).

9. Akashi, K., Traver, D., Miyamoto, T. & Weissman, I.L. A clonogenic common myeloid progenitor that gives rise to all myeloid lineages. *Nature* **404**, 193-197 (2000).

10. Kondo, M., Weissman, I.L. & Akashi, K. Identification of clonogenic common lymphoid progenitors in mouse bone marrow. *Cell* **91**, 661-672 (1997).

11. Inlay, M.A. *et al.* Ly6d marks the earliest stage of B-cell specification and identifies the branchpoint between B-cell and T-cell development. *Genes Dev* **23**, 2376-2381 (2009).

12. Hardy, R.R., Carmack, C.E., Shinton, S.A., Kemp, J.D. & Hayakawa, K. Resolution and characterization of pro-B and pre-pro-B cell stages in normal mouse bone marrow. *J Exp Med* **173**, 1213-1225 (1991).

13. Blasius, A.L., Barchet, W., Cella, M. & Colonna, M. Development and function of murine B220+CD11c+NK1.1+ cells identify them as a subset of NK cells. *J Exp Med* **204**, 2561-2568 (2007).

14. Asselin-Paturel, C. *et al.* Mouse type I IFN-producing cells are immature APCs with plasmacytoid morphology. *Nat Immunol* **2**, 1144-1150 (2001).

15. Chan, C.W. *et al.* Interferon-producing killer dendritic cells provide a link between innate and adaptive immunity. *Nat Med* **12**, 207-213 (2006).

16. Taieb, J. *et al.* A novel dendritic cell subset involved in tumor immunosurveillance. *Nat Med* **12**, 214-219 (2006).

17. Rumfelt, L.L., Zhou, Y., Rowley, B.M., Shinton, S.A. & Hardy, R.R. Lineage specification and plasticity in CD19- early B cell precursors. *J Exp Med* **203**, 675-687 (2006).

18. Godfrey, D.I., Kennedy, J., Suda, T. & Zlotnik, A. A developmental pathway involving four phenotypically and functionally distinct subsets of CD3-CD4-CD8- triple-negative adult mouse thymocytes defined by CD44 and CD25 expression. *J Immunol* **150**, 4244-4252 (1993).

19. Allman, D. *et al.* Resolution of three nonproliferative immature splenic B cell subsets reveals multiple selection points during peripheral B cell maturation. *J Immunol* **167**, 6834-6840 (2001).

20. Loder, F. *et al.* B cell development in the spleen takes place in discrete steps and is determined by the quality of B cell receptor-derived signals. *J Exp Med* **190**, 75-89 (1999).

21. Schlissel, M.S., Corcoran, L.M. & Baltimore, D. Virus-transformed pre-B cells show ordered activation but not inactivation of immunoglobulin gene rearrangement and transcription. *J Exp Med* **173**, 711-720 (1991).

22. Schlissel, M.S. & Baltimore, D. Activation of immunoglobulin kappa gene rearrangement correlates with induction of germline kappa gene transcription. *Cell* **58**, 1001-1007 (1989).

23. Inlay, M., Alt, F.W., Baltimore, D. & Xu, Y. Essential roles of the kappa light chain intronic enhancer and 3' enhancer in kappa rearrangement and demethylation. *Nat Immunol* **3**, 463-468 (2002).

24. Jurado, S. *et al.* The Zinc-finger protein ASCIZ regulates B cell development via DYNLL1 and Bim. *J Exp Med* **209**, 1629-1639 (2012).

25. Angelin-Duclos, C. & Calame, K. Evidence that immunoglobulin VH-DJ recombination does not require germ line transcription of the recombining variable gene segment. *Mol Cell Biol* **18**, 6253-6264 (1998).

26. Xiang, Y. & Garrard, W.T. The Downstream Transcriptional Enhancer, Ed, positively regulates mouse Ig kappa gene expression and somatic hypermutation. *J Immunol* **180**, 6725-6732 (2008).

27. LeBleu, V. *et al.* Stem cell therapies benefit Alport syndrome. *J Am Soc Nephrol* **20**, 2359-2370 (2009).

28. Jackson, A., Kondilis, H.D., Khor, B., Sleckman, B.P. & Krangel, M.S. Regulation of T cell receptor beta allelic exclusion at a level beyond accessibility. *Nature Immunology* **6**, 189-197 (2005).

29. Kehry, M.R. & Castle, B.E. Regulation of CD40 ligand expression and use of recombinant CD40 ligand for studying B cell growth and differentiation. *Semin Immunol* **6**, 287-294 (1994).

30. Schwickert, T.A. *et al.* Stage-specific control of early B cell development by the transcription factor Ikaros. *Nat Immunol* **15**, 283-293 (2014).

31. Revilla, I.D.R. *et al.* The B-cell identity factor Pax5 regulates distinct transcriptional programmes in early and late B lymphopoiesis. *EMBO J* **31**, 3130-3146 (2012).

32. Smeenk, L. *et al.* Molecular role of the PAX5-ETV6 oncoprotein in promoting B-cell acute lymphoblastic leukemia. *EMBO J* **36**, 718-735 (2017).

33. Jensen, C.T. *et al.* Dissection of progenitor compartments resolves developmental trajectories in B-lymphopoiesis. *J Exp Med* **215**, 1947-1963 (2018).

34. Farley, F.W., Soriano, P., Steffen, L.S. & Dymecki, S.M. Widespread recombinase expression using FLPeR (flipper) mice. *Genesis* **28**, 106-110 (2000).

35. McCormack, M.P., Forster, A., Drynan, L., Pannell, R. & Rabbitts, T.H. The LMO2 T-cell oncogene is activated via chromosomal translocations or retroviral insertion during gene therapy but has no mandatory role in normal T-cell development. *Mol Cell Biol* **23**, 9003-9013 (2003).

36. Phan, T.G. *et al.* B cell receptor-independent stimuli trigger immunoglobulin (Ig) class switch recombination and production of IgG autoantibodies by anergic self-reactive B cells. *J Exp Med* **197**, 845-860 (2003).

37. Langmead, B. & Salzberg, S.L. Fast gapped-read alignment with Bowtie 2. *Nat Methods* **9**, 357-359 (2012).

38. Ramirez, F., Dundar, F., Diehl, S., Gruning, B.A. & Manke, T. deepTools: a flexible platform for exploring deep-sequencing data. *Nucleic Acids Res* **42**, W187-191 (2014).

39. Schneider, C.A., Rasband, W.S. & Eliceiri, K.W. NIH Image to ImageJ: 25 years of image analysis. *Nat Methods* **9**, 671-675 (2012).

40. Anderson, S.J., Abraham, K.M., Nakayama, T., Singer, A. & Perlmutter, R.M. Inhibition of T-cell receptor beta-chain gene rearrangement by overexpression of the non-receptor protein tyrosine kinase p56lck. *EMBO J* **11**, 4877-4886 (1992).

41. Wojciechowski, J., Lai, A., Kondo, M. & Zhuang, Y. E2A and HEB are required to block thymocyte proliferation prior to pre-TCR expression. *J Immunol* **178**, 5717-5726 (2007).

42. Abe, K. *et al.* Novel lymphocyte-independent mechanisms to initiate inflammatory arthritis via bone marrow-derived cells of Ali18 mutant mice. *Rheumatology (Oxford)* **47**, 292-300 (2008).

43. Livak, K.J. & Schmittgen, T.D. Analysis of relative gene expression data using real-time quantitative PCR and the 2(-Delta Delta C(T)) Method. *Methods* **25**, 402-408 (2001).

44. Dobin, A. *et al.* STAR: ultrafast universal RNA-seq aligner. *Bioinformatics* **29**, 15-21 (2013).

45. Liao, Y., Smyth, G.K. & Shi, W. featureCounts: an efficient general purpose program for assigning sequence reads to genomic features. *Bioinformatics* **30**, 923-930 (2014).

46. Liao, Y., Smyth, G.K. & Shi, W. The R package Rsubread is easier, faster, cheaper and better for alignment and quantification of RNA sequencing reads. *Nucleic Acids Res* **47**, e47 (2019).

47. Robinson, M.D., McCarthy, D.J. & Smyth, G.K. edgeR: a Bioconductor package for differential expression analysis of digital gene expression data. *Bioinformatics* **26**, 139-140 (2010).

48. Chen, Y., Lun, A.T. & Smyth, G.K. From reads to genes to pathways: differential expression analysis of RNA-Seq experiments using Rsubread and the edgeR quasi-likelihood pipeline. *F1000Res* **5**, 1438 (2016).

49. Bolger, A.M., Lohse, M. & Usadel, B. Trimmomatic: a flexible trimmer for Illumina sequence data. *Bioinformatics* **30**, 2114-2120 (2014).

50. Li, H. Aligning sequence reads, clone sequences and assembly contigs with BWA-MEM. Preprint at https://arXiv:1303.3997v2; 2013.

51. Zhang, Y. *et al.* Model-based analysis of ChIP-Seq (MACS). *Genome Biol* **9**, R137 (2008).

52. Zhu, L.J. *et al.* ChIPpeakAnno: a Bioconductor package to annotate ChIP-seq and ChIP-chip data. *BMC Bioinformatics* **11**, 237 (2010).

53. Liao, Y., Smyth, G.K. & Shi, W. The Subread aligner: fast, accurate and scalable read mapping by seed-and-vote. *Nucleic Acids Res* **41**, e108 (2013).

54. Thomas-Claudepierre, A.S. *et al.* Mediator facilitates transcriptional activation and dynamic long-range contacts at the IgH locus during class switch recombination. *J Exp Med* **213**, 303-312 (2016).

55. D'Addabbo, P., Scascitelli, M., Giambra, V., Rocchi, M. & Frezza, D. Position and sequence conservation in Amniota of polymorphic enhancer HS1.2 within the palindrome of IgH 3'Regulatory Region. *BMC Evol Biol* **11**, 71 (2011).

56. Chakraborty, T. *et al.* A 220-nucleotide deletion of the intronic enhancer reveals an epigenetic hierarchy in immunoglobulin heavy chain locus activation. *J Exp Med* **206**, 1019-1027 (2009).

57. Ebert, A. *et al.* The distal V(H) gene cluster of the Igh locus contains distinct regulatory elements with Pax5 transcription factor-dependent activity in pro-B cells. *Immunity* **34**, 175-187 (2011).
