# Supplementary material for: An Erg-driven transcriptional program controls B cell lymphopoiesis
Source: Nat Commun. 2020 Jun 15;11:3013. doi: 10.1038/s41467-020-16828-y (PMC7296042; doi:10.1038/s41467-020-16828-y)
Supplement: Supplementary file 3 — Reporting Summary [file 41467_2020_16828_MOESM3_ESM.pdf]

nature research

Corresponding author(s): Ng, AP

Last updated by author(s): Apr 28, 2020

Reporting Summary

Nature Research wishes to improve the reproducibility of the work that we publish. This form provides structure for consistency and transparency in reporting. For further information on Nature Research policies, see Authors & Referees and the Editorial Policy Checklist.

Statistics

For all statistical analyses, confirm that the following items are present in the figure legend, table legend, main text, or Methods section.

n/a	Confirmed	
☐	☒	The exact sample size (n) for each experimental group/condition, given as a discrete number and unit of measurement
☐	☒	A statement on whether measurements were taken from distinct samples or whether the same sample was measured repeatedly
☐	☒	The statistical test(s) used AND whether they are one- or two-sided<br>*Only common tests should be described solely by name; describe more complex techniques in the Methods section.*
☐	☒	A description of all covariates tested
☐	☒	A description of any assumptions or corrections, such as tests of normality and adjustment for multiple comparisons
☐	☒	A full description of the statistical parameters including central tendency (e.g. means) or other basic estimates (e.g. regression coefficient) AND variation (e.g. standard deviation) or associated estimates of uncertainty (e.g. confidence intervals)
☐	☒	For null hypothesis testing, the test statistic (e.g. F, t, r) with confidence intervals, effect sizes, degrees of freedom and P value noted<br>*Give P values as exact values whenever suitable.*
☐	☒	For Bayesian analysis, information on the choice of priors and Markov chain Monte Carlo settings
☒	☐	For hierarchical and complex designs, identification of the appropriate level for tests and full reporting of outcomes
☒	☐	Estimates of effect sizes (e.g. Cohen's d, Pearson's r), indicating how they were calculated

Our web collection on statistics for biologists contains articles on many of the points above.

Software and code

Policy information about availability of computer code

Data collection	Flow cytometry; BD FACS LSRII, RNA-seq (Illumina); HiSeq 2500, ChIP-seq and ATAC-seq; NextSeq 500 (Illumina), HiC; MiSeq (Illumina). Fluorescence In Situ Hybridisation; LSM 880 confocal microscope (Zeiss).
Data analysis	Flow collection software: BD FACSDiva 7 (BD Biosciences). Analysis software: all data were imported as uncompensated files and re-compensated and analysed on FlowJo 8 (FlowJo Tree Star RRID:SCR_008520), Excel for Mac v16 (Microsoft), Prism v8 (GraphPad).<br><br>Fluorescence In Situ Hybridisation software Zen 2.3 (Zeiss Microscopy) RRID:SCR_013672 was used for data collection. Analysis was undertaken using ImageJ RRID:SCR_003070, Huygens Software Scientific Volume Imaging RRID:SCR_014237.<br><br>Bioinformatic analysis was performed using: R Studio RStudio, Inc RRID:SCR_000432, R R Project for Statistical Computing RRID:SCR_001905, R packages Rsubread, limma, edgeR, Rmagic, Rtsne, csaw, diffHic, InterationSet, Sushi Bioconductor RRID:SCR_001905, MACS2 Liu Lab Harvard University RRID:SCR_013291, Bowtie2 RRID:SCR_005476, deepTools RRID:SCR_016366, R packages pheatmap viridis CRAN RRID:SCR_003005S, MAGIC Krishnaswamy Lab Yale University (https://github.com/pkathail/magic/).

For manuscripts utilizing custom algorithms or software that are central to the research but not yet described in published literature, software must be made available to editors/reviewers. We strongly encourage code deposition in a community repository (e.g. GitHub). See the Nature Research guidelines for submitting code & software for further information.

Data

Policy information about availability of data

All manuscripts must include a data availability statement. This statement should provide the following information, where applicable:

- Accession codes, unique identifiers, or web links for publicly available datasets
- A list of figures that have associated raw data
- A description of any restrictions on data availability

The following datasets analysed in the current study are available at the NCBI Gene Expression Omnibus, accession GSE132852 [https://www.ncbi.nlm.nih.gov/geo/

query/acc.cgi?acc=GSE132852] (ATAC-seq), GSE132853 [https://www.ncbi.nlm.nih.gov/geo/query/acc.cgi?acc=GSE132853] (ChIP-seq), GSE132854 [https://www.ncbi.nlm.nih.gov/geo/query/acc.cgi?acc=GSE132854] (RNA-seq), GSE133246 [https://www.ncbi.nlm.nih.gov/geo/query/acc.cgi?acc=GSE133246] (Hi-C).
Other publicly available datasets are available at at the NCBI Gene Expression Omnibus, accession GSM1296532, GSM1296537 (ChIP-seq Ebf1), GSM1296537 (ProB_Rag2_input), GSM932924 (ChIP-seq Pax5), GSM1145867 (ProB_Rag2_Input_2), GSM2255547 (ChIP-seq H3K4me3), GSM2255552 (ChIP-seq H3K27ac), GSM2879293, GSM2879294, GSM2879295 (RNA-seq Ebf1 knockout), GSM2879296, GSM2879297, GSM2879298 (RNA-seq Pax5 knockout), GSM2879299, GSM2879300, GSM2879301 (RNA-seq wild-type), GSE114793 (scRNA-seq wild-type).
The source data underlying Fig. 1b, 1d, 1e, 1f, 2a, 2b, 2d, 3a, 3b, 3c, 3d, 3f, 3g, 4d, 5b, 5d, 5e, 5f, Supplementary Fig. 2a, 2b, 2c, 3b, 3c are provided as Source Data file. The data supporting this study are available in the Article, Supplementary Information, Source Data or available from the authors upon reasonable requests.

Field-specific reporting

Please select the one below that is the best fit for your research. If you are not sure, read the appropriate sections before making your selection.

☒ Life sciences ☐ Behavioural & social sciences ☐ Ecological, evolutionary & environmental sciences

For a reference copy of the document with all sections, see nature.com/documents/nr-reporting-summary-flat.pdf

Life sciences study design

All studies must disclose on these points even when the disclosure is negative.

Sample size	Sample sizes were chosen for matched sample analysis to control for an alpha of 0.05 and beta of 0.20 using a P-value significance threshold of 0.05.
Data exclusions	No data were excluded.
Replication	All experiments were replicated at least twice (as indicated in figure legends).<br>All attempts at replicating the observations described in this manuscript were successful.
Randomization	Mice and samples were randomly allocated to each analysis group at the start of experiment.
Blinding	Blinding was performed by removal of identifying data from each sample while primary data was collected during an experiment. Once data had been collected blinding was not required for statistical or bioinformatic analysis as objective readouts had been used in all experiments and all samples were analyzed using an identical method for each experiment.

Reporting for specific materials, systems and methods

We require information from authors about some types of materials, experimental systems and methods used in many studies. Here, indicate whether each material, system or method listed is relevant to your study. If you are not sure if a list item applies to your research, read the appropriate section before selecting a response.

Materials & experimental systems

n/a	Involved in the study	
☐	☒	Antibodies
☒	☐	Eukaryotic cell lines
☒	☐	Palaeontology
☐	☒	Animals and other organisms
☒	☐	Human research participants
☒	☐	Clinical data

Methods

n/a	Involved in the study	
☐	☒	ChIP-seq
☐	☒	Flow cytometry
☒	☐	MRI-based neuroimaging

Antibodies

Antibodies used

Antibodies for Western blot/ChIP:
Anti-Erg Santa Cruz Biotechnology sc-354, Anti-Erg Abcam ab133264 (used 1:1000 for Western blot), Anti-Ebf1 Abcam ab108369 (used 1:1000 for Western blot), Anti-Pax5 In-house Clone: 1H9 (used 1:100 for Western blot).

Flow Cytometry Antibodies:
Ter119 APC-Cy7 1:150 BD Biosciences Clone : Ly-76, CD41 PE 1:333 BD Biosciences Clone : MWReg30, Gr1 PE-Cy7 1:333 BD Biosciences Clone :Ly6G &Ly6C, Mac1 Alexa700 1:333 BD Biosciences Clone : CD11b, NK1.1 PerCPCy5_5 1:333 BD Biosciences Clone : N418, CD45R/B220 APC 1:333 BD Biosciences Clone : RA3-6B2, CD19 Biotin 1:333 BD Biosciences Clone : 1D3, CD3 PE 1:333 BD Biosciences Clone : 17A2, CD4 APC 1:300 BD Biosciences Clone : CK1.5, CD8a PE 1:300 BD Biosciences Clone : 53.6.7. Sca1 (Ly6A/E) PE-Cy7 1:200 BD Biosciences Clone : D7, cKit (CD117) PerCP-Cy5.5 1:200 BD Biosciences Clone : 2B8 or ACK4, CD150 APC 1:100 Biolegend Clone : TC15-12F12.2, CD105 PE 1:100 BD Biosciences Clone : MJ7/18, CD16/32 PE-Cy7 1:100 BD Biosciences Clone ; 24G2, CD127 Biotin 1:100 eBioscience Clone : A7R34, CD135 PE 1:50 Biolegend Clone : A2F10, Ly6D APC 1:1000 BD Biosciences Clone : 49-H4, CD21/35 FITC 1:400 BD Biosciences Clone : 7G6, CD23 PE-Cy7 1:400 BD Biosciences Clone : B3B4, CD93 PerCP-Cy5.5 1:400 BD Biosciences Clone : AA4.1, CD24 PerCP-Cy5.5 1:400 BD Biosciences Clone : M1/69, CD43 PE-

	Cy7 1:400 BD Biosciences Clone : S7, CD45.2 Biotin 1:400 BD Biosciences Clone : S450-15-2, CD45.1 Alexa700 1:400 BD Biosciences Clone : A20, IgMb APC-Cy7 1:150 BD Biosciences Clone : AF6-78, IgD PE 1:333 BD Biosciences Clone : 11-26c.2a, CD138 PE 1:200 BD Biosciences Clone : 281.2, IgG1 APC 1:200 BD Biosciences Clone : X56, CD25 PerCP-Cy5.5 1:400 BD Biosciences Clone : 3C7, CD44 PE-Cy7 1:300 BD Biosciences Clone : IM7
Validation	All antibodies were validated by manufacturers for the applications and species used in this study and were supported by publications or internal validation. See manufacturers websites for validation statements (www.biolegend.com; www.bdbiosciences.com; www.ebioscience.com; https://www.abcam.com) found in technical data sheets.<br>All antibodies were tested by titration including matching isotype controls against known reacting cell types in parallel.<br>All experiments included appropriate negative and positive control populations.

Animals and other organisms

Policy information about studies involving animals; ARRIVE guidelines recommended for reporting animal research

Laboratory animals	All mice were 6 to 18 weeks at start of experiment. Both male and female mice were used. Mice strains used were bred on a C57BL/6J background. Mice were co-housed in a barrier facility and analysed from 6 to 18 weeks of age. Euthanasia was performed by CO2 induction or cervical dislocation.<br><br>Mice carrying the Ergtm1a(KOMP)wtsi knock-first reporter allele (ErgKI, KOMP Knockout Mouse Project) were generated by gene targeting in ES cells. Mice with a conditional Erg knockout allele (Ergfl) from which the IRES-LacZ cassette was excised were generated by interbreeding ErgKI mice with Flpe transgenic mice. Rag1Cre mice, in which Cre recombinase is expressed during lymphopoiesis from the CLP stage, were interbred with Ergfl mice to generate mice lacking Erg in lymphopoiesis (Rag1CreT/+;ErgΔ/Δ) and Rag1Cre+/+;Ergfl/fl (Ergfl/fl) controls.<br><br>Mice carrying the rearranged immunoglobulin heavy chain IgHVH10tar allele were a gift from Professor Robert Brink.<br><br>The cEμΔ/Δ and μAΔ/Δ mice were generated by the MAGEC laboratory (Walter and Eliza Hall Institute of Medical Research) on a C57BL/6J background. To generate cEμΔ mice, 20 ng/μl of Cas9 mRNA, 10 ng/μl of sgRNA (GTTGAGGATTCAGCCGAAAC and ATGTTGAGTTGGAGTCAAGA) and 40 ng/μl of oligo donor (CAAGCTAAAATTAAAAGGTTGAACTCAATAAGTTAAAAGAGGACCTCTCCAGTTTCGGCTCAACTCAACATTGCTCAATTCATTTAAAAATATTTGAAACTTAATTTATTATTGTTAAAA) were injected into the cytoplasm of fertilized one-cell stage embryos. To generate μAΔ mice, 20 ng/μl of Cas9 mRNA, 10 ng/μl of sgRNA (GAACACCTGCAGCAGCTGGC) and 40 ng/μl of oligo donor (GCTACAAGTTTACCTAGTGGTTTTATTTTCCCTTCCCCAAATAGCCTTGCCACATGACCTGCCAGCTGCTGCAGGTGTTCTGGTTCTGATCGGCCATCTTGACTCCAACTCAACATTGCT) were injected into the cytoplasm of fertilized one-cell stage embryos. Twenty-four hours later, two-cell stage embryos were transferred into the oviducts of pseudo-pregnant female mice. Viable offspring were genotyped by next-generation sequencing.
Wild animals	The study did not involve wild animals.
Field-collected samples	The study did not involve field collected samples.
Ethics oversight	This study was performed in accordance with the Australian Code for the Care and Use of Animals for Scientific Purposes, published by the Australian National Health and Medical Research Council. Experimental procedures were approved by the Walter and Eliza Hall Institute of Medical Research Animal Ethics Committee.

Note that full information on the approval of the study protocol must also be provided in the manuscript.

ChIP-seq

Data deposition

☒ Confirm that both raw and final processed data have been deposited in a public database such as GEO.

☒ Confirm that you have deposited or provided access to graph files (e.g. BED files) for the called peaks.

Data access links<br>*May remain private before publication.*	These data have been deposited in Gene Expression Omnibus database (accession number GSE132853 [https://www.ncbi.nlm.nih.gov/geo/query/acc.cgi?acc=GSE132853]).
Files in database submission	GSM3895107, GSM3895108
Genome browser session (e.g. UCSC)	No longer applicable

Methodology

Replicates	ChIP-seq was performed on C57BL/6J proB cells with Rag1CreT/+;ErgΔ/Δ thymocytes as a negative control.<br>Confirmatory replicates were performed on C57BL/6J, μA Δ/Δ proB cells and Rag1CreT/+;ErgΔ/Δ pre-proB cells as negative controls (see Supplementary Figure 4b).
Sequencing depth	Each library was sequenced using a high output single end 75 base pair kit on the NextSeq500 (Illumina) to a minumum of ~9.3 million read depth with a minimum of ~9 million uniquely mapped reads per library.

Antibodies	Anti-Erg Santa Cruz Biotechnology sc-354, Anti-Erg Abcam ab133264 (confirmatory analysis).
Peak calling parameters	Peaks were called using MACS2 with default parameters to identify peaks using C17 antibody for Erg binding (GSM3895107) with Rag1CreT/+;ErgΔ/Δ thymocytes (GSM3895108) as a negative control to filter peaks not due to Erg binding.
Data quality	We undertook strand cross-correlation analysis to estimate the fragment length and to ensure the immuno-precipitated peak was higher than phantom peak in all experiments indicating that the pull down was successful. We identified 2,699 peaks with a FDR < 0.05 and 2-fold enrichment.
Software	DNA reads were adapter trimmed using Trimmomatic and aligned to the GRCm38/mm10 build of the Mus musculus genome using the BWA aligner. Peaks were called using MACS2.

Flow Cytometry

Plots

Confirm that:

☒ The axis labels state the marker and fluorochrome used (e.g. CD4-FITC).

☒ The axis scales are clearly visible. Include numbers along axes only for bottom left plot of group (a 'group' is an analysis of identical markers).

☒ All plots are contour plots with outliers or pseudocolor plots.

☒ A numerical value for number of cells or percentage (with statistics) is provided.

Methodology

Sample preparation	Single-cell suspensions from bone marrow, lymph node or spleen were prepared in balanced salt solution (BSS-CS: 0.15M NaCl, 4mM KCl, 2mM CaCl2, 1mM MgSO4, 1mM KH2PO4, 0.8mM K2HPO4, and 15mM HEPES supplemented with 2% [vol/vol] bovine calf serum). Analysis of blood was performed after erythrocyte lysis in buffered 156mM NH4Cl. Staining was performed using biotinylated or fluorochrome-conjugated antibodies specific for murine antigens Ter119 (Ly-76), CD41 (MWReg30), Gr1 (Ly6G and Ly6C), Mac1 (CD11b), NK1.1, CD11c (N418), CD45R/B220 (RA3-6B2), CD19 (1D3), CD3 (17A2), CD4 (GK1.5), CD8a (53.6.7), Sca1 (Ly6A/E, D7), cKit (CD117, ACK4 or 2B8), CD150 (TC15-12F12.2), CD105 (MJ7/18), CD16/32 (24G2), CD127 (A7R34), CD135 (A2F10), Ly6D (49-H4), CD21/CD35 (7G6), CD23 (B3B4), CD93 (AA4.1), CD24 (M1/69), CD43 (S7), CD45.2 (S450-15-2), CD45.1 (A20), IgMb (AF6-78), IgD (11-26c.2a), CD138 (281.2), IgG1 (X56), CD25 (3C7), CD44 (IM7). Secondary staining used streptavidin PE-Texas-Red (Invitrogen). See Supplementary Table 4 for antibody dilutions and catalogue numbers for commercial antibodies. FACS-Gal analysis was performed using warm hypotonic loading of fluorescein di β-D-galactopyranoside (Molecular Probes) on single cells followed by immunophenotyping using relevant surface antigens as defined in Supplementary Table 1.
Instrument	Cells were analyzed using a LSR II (Becton Dickinson)
Software	Data was collected using BD FACSDiva 7 (BD Biosciences) software and uncompensated data exported and re-analysed using FlowJo software (Version 8.8.7, Tree Star).
Cell population abundance	Cell population abundance: (1) Ergfl/fl or Rag1CreT/+;ErgΔ/Δ contained >80% B220+ bone marrow after positive selection for sorting pre-proB, proB or preB populations (2) MSCV-transduced lineage negative bone marrow cultured on OP9 stroma with IL7 and Flt3L demonstrated >50% GFP expressing cells for sorting. Cell purity was validated > 90% by sample re-analysis.
Gating strategy	FSC-pulse width versus SSC-H; SSC-pulse width versus FSC-H; FSC-A versus SSC-A, fluoro-gold for live mononuclear cells.

☒ Tick this box to confirm that a figure exemplifying the gating strategy is provided in the Supplementary Information.
