# Supplementary material for: An Erg-driven transcriptional program controls B cell lymphopoiesis
Source: Nat Commun. 2020 Jun 15;11:3013. doi: 10.1038/s41467-020-16828-y (PMC7296042; doi:10.1038/s41467-020-16828-y)
Supplement: Supplementary file 4 — Description of Additional Supplementary Files [file 41467_2020_16828_MOESM4_ESM.pdf]

Title: Supplementary Data 1

Description: RNA-seq for differentially expressed genes. Rag1Cre$^{T/+}$;Erg$^{\Delta/\Delta}$ and Erg$^{fl/fl}$ pre-proB cells, Ebf1$^{\Delta/\Delta}$ pre-proB cells and Pax5$^{\Delta/\Delta}$ proB cells and wild-type, Rag1Cre$^{T/+}$;Erg$^{\Delta/\Delta}$ pre-proB cells and Rag1Cre$^{T/+}$;Erg$^{\Delta/\Delta}$ pre-proB transduced with MSCV-Ebf1 and MSCV-Pax5 retroviruses. Differentially expressed genes between Rag1Cre$^{T/+}$;Erg$^{\Delta/\Delta}$ and Erg$^{fl/fl}$ pre-proB cells, OP9 cultured WT, Ebf1$^{\Delta/\Delta}$ pre-proB cells and Pax5$^{\Delta/\Delta}$ proB cells, and OP9 cultured B220^{+} C57BL/6 proB, Rag1Cre$^{T/+}$;Erg$^{\Delta/\Delta}$ pre-proB and Rag1Cre$^{T/+}$;Erg$^{\Delta/\Delta}$ cells transduced with MSCV-Ebf1 and MSCV-Pax5 retroviruses. **See Fig. 4, Fig. 5**.

Title: Supplementary Data 2

Description: ChIP-seq for the Erg, Ebf1 and Pax5 gene regulatory network. Erg, Ebf1 and Pax5 ChIP binding coordinates to differentially expressed genes in Rag1Cre$^{T/+}$;Erg$^{\Delta/\Delta}$ pre-proB cells. **See Fig. 6.**
